# When are Uncontrolled Trials in TB Scientifically and Ethically Justified: Borrowing Wisdom From Early AIDS Clinical Trials

**DOI:** 10.1093/cid/ciag175

**Published:** 2026-03-13

**Authors:** Lindsay McKenna, Mike Frick, Valentina A Alarcón-Guizado, Geraint R Davies, Jennifer J Furin, Lorenzo Guglielmetti, Norbert Heinrich, Marcus Low, Mariama Mahmoud, Vidya Mave, Norbert Ndjeka, Patrick P J Phillips, Samuel Schumacher, Mark Harrington, Carole D Mitnick

**Affiliations:** Tuberculosis Project, Treatment Action Group, New York, New York, USA; Tuberculosis Project, Treatment Action Group, New York, New York, USA; Dirección de Prevención y Control de Tuberculosis, Dirección General de Intervenciones Estratégicas en Salud Pública, Ministerio de Salud, Lima, Peru; Department of Clinical Infection, Microbiology and Immunology, Institute of Infection, Veterinary and Ecological Sciences, University of Liverpool, Liverpool, United Kingdom; Department of Global Health and Social Medicine, Harvard Medical School, Boston, Massachusetts, USA; Department of Infectious, Tropical Diseases and Microbiology, IRCCS Sacro Cuore Don Calabria Hospital, Negrar di Valpolicella, Verona, Italy; German Center for Infection Research, Munich Partner Site, Institute of Infectious Diseases and Tropical Medicine, LMU University Hospital, LMU Munich, Munich, Germany; Fraunhofer Institute for Translational Medicine and Pharmacology ITMP, Immunology, Infection and Pandemic Research, Munich, Germany; Spotlight, Cape Town, South Africa; National Leprosy and Tuberculosis Control Program, Ministry of Health, Freetown, Sierra Leone; Center for Infectious Diseases in India, Johns Hopkins India, Pune, India; School of Medicine, Division of Infectious Diseases, Johns Hopkins University, Baltimore, Maryland, USA; National Department of Health, Pretoria, South Africa; University of California SanFrancisco Center for Tuberculosis, University of California San Francisco, San Francisco, California, USA; Global Programme on Tuberculosis and Lung Health, World Health Organization, Geneva, Switzerland; Treatment Action Group, New York, New York, USA; Department of Infectious, Tropical Diseases and Microbiology, IRCCS Sacro Cuore Don Calabria Hospital, Negrar di Valpolicella, Verona, Italy

**Keywords:** tuberculosis, evidence, randomized, internally controlled trials, high evidentiary standards

## Abstract

Tuberculosis treatment trials have used various comparison strategies. To inform trial design for novel tuberculosis regimens, we applied an existing framework for evaluating when uncontrolled trials might be justified. We conclude that the conditions are not met for tuberculosis treatment and that uncontrolled phase 3 trials should not be performed.

In 1990, when HIV-1 care and research were undergoing revolutionary changes, Byar et al published a paper describing design considerations for HIV clinical trials, including 5 requirements for justifying the use of uncontrolled trials (Box) [1]. It is timely to apply these requirements to tuberculosis (TB). Trials have recently changed global standards of care for TB treatment notably for multidrug-/rifampin-resistant TB (MDR/RR-TB). Studies have used a variety of strategies for comparison, ranging from no to internally controlled comparators comprising regimen(s) that evolved alongside normative guidance during the trial (Table 1). A new wave of phase 3 trials will soon advance the next generation of TB treatment regimens. While randomized, controlled trials (RCTs) remain the keystone of evidence-based healthcare, here we consider when uncontrolled Phase 3 trials of TB treatment may be justified according to: the Byar et al requirements, current ethical standards, and new World Health Organization (WHO) guidance [1–3]. Both Byar et al and WHO guidance limit the use of uncontrolled studies to “special situations” (Byar) or “exceptional circumstances” (WHO) [1, 3].

**Table 1.** Tuberculosis Treatment Trial Design Trade-offs

Design	Motivation	Challenges	Example Trial Name: Clinicaltrials.gov Identifier
Uncontrolled study	Faster, smaller, simpler, cheaper up-front; SoC questionable	Inference difficult; likely low certainty evidence; requires more research with additional, deferred costs.	Nix-TB: NCT02333799
RCT + exploratory arm for specific population (eg, uncontrolled MDR/RR-TB arm in a DS-TB RCT)	In priority DS-TB population, motivations are same as below. For uncontrolled group, motivation is same as above; offers benefit of proof-of-concept to de-risk larger follow-on in the non-priority, MDR/RR-TB population.	For uncontrolled arm: Same as above; possible poor generalizability. In addition, it is hard to translate benefit-harm balance from controlled (DS) to uncontrolled (MDR/RR) populations.	SimpliciTB: NCT03338621 STAND: NCT02342886 PAN-TB: NCT05971602
RCT with internal concurrent control	Reduces several biases; permits conclusions about performance in relation to existing SoC; likely higher certainty of evidence.	Larger, slower; more expensive up-front; control may comprise evolving standard of care.*	STREAM: NCT02409290; Study 31: NCT02410772; NEXT*: NCT02454205; MDR-END: NCT02619994; TB-PRACTECAL*: NCT02589782; endTB*: NCT02754765; BEAT-Tuberculosis: NCT04062201c endTB-Q*: NCT03896685

Abbreviations: MDR/RR-TB, rifampicin/multidrug-resistant TB; RCT, randomized controlled trial; SoC, standard of care; DS-TB, drug-susceptible TB.

Box 1 Five requirements justifying uncontrolled trials from Byar et al (1990) [1]:

“[…] there are special situations in which the use of an uncontrolled phase III clinical trial to evaluate the efficacy of a new drug may be justified. We believe that such special situations must meet all the following requirements:

1. There must be no other treatment appropriate to use as a control;
2. There must be sufficient experience to ensure that the patients not receiving therapy will have a uniformly poor prognosis;
3. The therapy must not be expected to have substantial side effects that would compromise the potential benefit to the patient;
4. There must be a justifiable expectation that the potential benefit to the patient will be sufficiently large to make interpretation of the results of a nonrandomized trial unambiguous;
5. The scientific rationale for the treatment must be sufficiently strong that a positive result would be widely accepted.”

## APPLICATION OF BYAR ET AL REQUIREMENTS TO MODERN TB TRIALS

There must be: (1) “no other treatment appropriate to use as a control” and (2) “sufficient experience to ensure that patients not receiving therapy will have a uniformly poor prognosis.” These are more logical in reverse order. With respect to (2), untreated TB has a 10-year case fatality of ∼70% [4]. As an airborne, infectious disease, it spreads through and harms communities. Criterion 1, mirrored in ethical standards [2], requires comparison unless no acceptable comparator exists. This condition is not met since WHO recommends: 2 treatments for drug-susceptible (DS) TB, as many as 6 for MDR/RR-TB, and 3 for fluoroquinolone-resistant MDR/RR-TB (pre-XDR-TB), all with reported efficacy exceeding 80% and death occurring in <5% [5–9]. For extensively drug-resistant TB (XDR-TB)—TB caused by bacteria resistant to key first- and second-line drugs (rifampin, fluoroquinolones, and bedaquiline or linezolid)—a comparator exists; its “appropriateness” is debatable. It comprises individualized, longer regimens composed with currently available oral and intravenous or injectable agents [5]. One recent small study revealed TB-free survival at 18 months in only 52% of 82 people with XDR-TB [10]. Another recorded favorable outcomes in only 13% (of 38 people) at end of treatment [11]. These studies suggest poor prognosis with standard XDR-TB treatment, but considerable uncertainty remains after only a few, small, nonrandomized studies have reported. In summary, conditions 1 and 2 cannot be met with confidence for TB of any resistance category.

(3) “The therapy must not be expected to have substantial side effects that would compromise potential benefit to the patient.” Requirement 3 demands confidence in the safety and tolerability of any experimental regimen to be tested without comparison. During DS-TB treatment, which has long been considered “safe and tolerable,” grade 3 or higher adverse events (severe AEs) occur in nearly 20% of people treated [6]. Severe AEs are even more common in the treatment of MDR/RR and pre-XDR-TB [7–9]. Newer members of extant drug classes, such as the diarylquinoline sorfequiline and the oxazolidinone sutezolid, and the development of long-acting delivery systems for anti-TB drugs offer potential for reduced toxicity. Novel products are now evaluated in combination sooner in the development process, possibly generating earlier evidence about regimen safety and tolerability. However, data from early clinical and preclinical studies of multidrug regimens have been insufficient to generate confidence in their safety and tolerability. Requirement 3 is also unlikely to be met.

(4) “There must be a justifiable expectation that the potential benefit to the patient will be sufficiently large to make interpretation of the results of a nonrandomized trial unambiguous.” And (5) “The scientific rationale for the treatment must be sufficiently strong that a positive result would be widely accepted.” Together, we interpret these requirements to mean that (1) the totality of evidence—preclinical activity; translational evidence including on dosing, duration, plasma PK/PD, lesion penetration; and phase 2 safety and efficacy data—is aligned, consistent, and supportive of the intervention; (2) accumulated evidence portends confidence about benefit; and (3) the anticipated benefit is large compared with an external standard. This is an appealing scenario. At present, however, there is no validated surrogate marker for clinical cure in TB. TB regimen development has seen a number of near misses where accumulated evidence from preclinical through phase 2 studies has supported a treatment innovation which failed to demonstrate non-inferiority to the existing standard of care in phase 3 [12, 13]. The expectation of a “large” benefit is challenged by 2 factors. First, “trial effect” improves performance of standard of care (SoC) and of interventions compared to their performance in routine care. In 3 recent MDR/RR-TB treatment trials, 4 experimental regimens achieved >75% treatment success while being not non-inferior to the internal comparator. In those trials, observed comparator response rates exceeded expected: 84.5% versus 70% in endTB; 59% versus 50% in TB-PRACTECAL; and 89% versus 75% in endTB-Q [7–9]. Without internal comparators, erroneous conclusions might have been drawn about the relative efficacy of these regimens. Lastly, the ability to anticipate a large benefit is limited by the ceiling of 100% efficacy. With WHO-endorsed treatments for drug-susceptible TB (DS-TB), MDR/RR-TB, and pre-XDR-TB all curing at least 85% of trial participants, the margin for efficacy improvement is narrow. XDR-TB outcomes still hold ample room for improvement. While not ready today, anticipated advances in translational models, through initiatives like the NIH-funded PreDicTR, and to biomarkers for TB treatment response, could increase confidence in prediction of an efficacy benefit.

## DISCUSSION

A diversity of approaches has been used for comparators in recent trials of TB treatments. They fall into 3 general categories (Table 1): (1) uncontrolled or historically controlled; (2) partially controlled: internally controlled for primary population, uncontrolled for other; (3) fully, internally controlled. Using recent experience in TB trials and anticipating trials that will advance the next generation of TB regimens, we explored whether the 5 Byar et al requirements for an uncontrolled study could be met [1]. We concluded that all 5 requirements could not be satisfied in any instance of a phase 3 trial of treatment for any form of TB at present. Trials to improve treatment of XDR-TB might meet requirements 1 and 2. Substantial improvements in the safety/tolerability of regimens and in the ability to predict Phase 3 trial results will be necessary to fulfill the other criteria.

Single-arm studies are promoted as an efficient, ethical approach for developing treatments for the most resistant forms of TB. Essentially asserting the primacy—and fulfillment—of requirements 1–3, the argument is that uncontrolled studies allow all participants to access new agents without risk of randomization to a standard rescue therapy, which carries major toxicities. This presupposes that trials are the only path to accessing experimental treatments when in fact, under most regulatory systems, compassionate use/expanded access mechanisms can provide access to those not best served by randomized trials [14]. The argument also disregards factors that undermine requirement 3. First, Byar et al warn that researchers often overestimate benefits and underestimate harms [1]. Second, the Helsinki Declaration demands continuous assessment of risks and burdens; the harm/benefit balance assessed at trial start is not immutable [2]. Internal controls permit safety monitoring committees to make real-time comparisons of safety and tolerability between treatment groups. Third, researchers and participants may value treatment characteristics differently. Researchers may deem a new TB regimen to have modest drawbacks relative to its benefits (eg, shortened treatment), but people treated for TB may express different priorities (eg, tolerability). Controlled trials uniquely capture comparable participant treatment experiences, information that is important for policy-making [3].

Single-arm studies are touted for being lower cost. However, relatively higher up-front costs, increased sample size of RCTs, and extended time required to complete those that utilize a longer control regimen, should be understood in relation to the long-term benefits of generating quality evidence for guidelines. Studies without internal controls may miss benefits (or harms) or characterize benefits (or harms) in a way that is sufficient only for a conditional recommendation. Per WHO, conditional recommendations result in the following: “substantial debate may be required before the policy is adopted by Member States; clinicians will need to discuss different management options with each patient; … and additional research would be likely to strengthen and possibly alter the recommendation” [3] (P.19). The cost of such additional TB research is often borne by public institutions, rarely by drug developers [15]. Another cost is delayed access for patients. Both costs were reflected in the cautious introduction of the landmark 6-month BPaL regimen for difficult-to-treat drug-resistant TB following the single-arm Nix-TB trial [16]. Notably, BPaL was recommended by WHO for routine use and widely adopted only after data became available from an RCT, TB-PRACTECAL [7].

Partially controlled trials have been used to estimate relative efficacy and safety in a primary population and absolute response in a secondary population. One phase 3 trial illustrates the limitations of this approach. SimpliciTB was designed to compare 4 months of an intervention to the 6-month standard of care for DS-TB. A parallel cohort of participants with MDR/RR-TB received the intervention for 6 months. Poor tolerability resulting in unacceptably high rates of unfavorable outcomes in DS-TB—16% in the experimental arm compared with 6.9% in the standard of care—ended regimen development activities for both DS-TB and MDR/RR-TB [17]. Benefit-harm balance, which may have been different for the latter group, could not be separately evaluated. Other internally controlled designs may serve as alternatives to the partially controlled trial and permit retention of the evidentiary benefits of a control while reducing exposure to it: an unequal allocation ratio puts fewer participants in the control arm (with a modest increase in sample size) while adaptive platform trials update the standard of care and experimental regimens over time.

Fundamentally, internal comparators are possible in TB treatment trials. Standardized regimens are now established for DS, MDR/RR, and pre-XDR-TB. A standard comprising individualized control regimens was previously used in trials of MDR/RR-TB and pre-XDR-TB treatment and can be used for XDR-TB trials. Trials with this comparator have informed policy despite the comparator's variable composition and evolution over time to align with improved standards [7–9]. Although results may be more complex to interpret with a heterogeneous standard than with a uniform one, use of the former strengthens evidence relative to having no or an outdated control. While its harm-benefit balance is not yet known, a standard for XDR-TB exists and the robust pipeline of new anti-TB agents offers potential for its improvement. Including the XDR-TB standard as a comparator in a randomized study would inform both the absolute performance of that treatment and its performance relative to a novel regimen.

Future trials may examine transformations in TB care—oral regimens comprising all new agents and/or dramatically shortened regimens containing long-acting injectables. Using uncontrolled studies to test these revolutionary changes could lead to profound delays in delivering health improvements to the intended beneficiaries—even if supported by a strong scientific rationale [3]. Although such evidence may be admitted by regulatory authorities and/or normative bodies, uncontrolled and partially controlled trials preclude drawing clear conclusions about the efficacy and safety of an intervention for the population in which it is studied. This may effectively compromise the terms of the compact between the study team and research volunteers about the value of the research [2].

Lastly, maintaining high evidentiary standards for TB treatment is important to upholding the right to science today and to preserving the precedent for the future [18]. Writing about the FDA approval of pretomanid as part of the BPaL regimen, the Global TB Community Advisory Board emphasized that evidentiary expediency today should “not inadvertently do a disservice to TB patients of the future” [19].

## CONCLUSION

Advances in TB treatment research have greatly improved standards of care, particularly for people with MDR/RR-TB. Although recent uncontrolled TB treatment studies have been influential, they occurred in an era when DR-TB treatment options were more restricted and the studies held limited value for TB treatment policy development. Applying the Byar et al requirements, we conclude that the 5 requirements justifying single-arm, uncontrolled studies cannot be met for phase 3 TB treatment trials. This includes treatment for MDR/RR-TB and XDR-TB. Fundamentally, people with these forms of disease have the same right as people with DS-TB to treatment based on quality evidence. It is incumbent on trialists to design trials that uphold evidentiary, ethical, and regulatory standards while providing timely results.

## References

[1] Byar DP, Schoenfeld DA, Green SB, et al Design considerations for AIDS trials. N Engl J Med 1990; 323:1343–8. 2215622 10.1056/NEJM199011083231912

[2] World Medical Association . *WMA Declaration of Helsinki – Ethical Principles for Medical Research Involving Human Participants*. WMA, **2024**. Available at: https://www.wma.net/policies-post/wma-declaration-of-helsinki/. Accessed 28 July 2025. 10.1001/jama.2024.21972 39425955

[3] World Health Organization . *Guidance on Evidence Generation on New Regimens for Tuberculosis Treatment*. WHO, **2024**. Available at: https://www.who.int/publications/i/item/9789240103627#:∼:text=The%20Guidance%20on%20evidence%20generation,novel%20regimens%20for%20TB%20treatment. Accessed 28 July 2025.

[4] Tiemersma EW, Van Der Werf MJ, Borgdorff MW, Williams BG, Nagelkerke NJD. Natural history of Tuberculosis: duration and fatality of untreated pulmonary Tuberculosis in HIV negative patients: a systematic review. PLoS One 2011; 6:e17601. 21483732 10.1371/journal.pone.0017601 PMC3070694

[5] World Health Organization . *WHO Consolidated Guidelines on Tuberculosis. Module 4: Treatment and Care*. WHO, **2025**. Available at: https://iris.who.int/bitstream/handle/10665/380799/9789240107243-eng.pdf?sequence=1. Accessed 28 July 2025. 40163610

[6] Dorman SE, Nahid P, Kurbatova EV, et al Four-month rifapentine regimens with or without moxifloxacin for tuberculosis. N Engl J Med 2021; 384:1705–18. 33951360 10.1056/NEJMoa2033400 PMC8282329

[7] Nyang’wa BT, Berry C, Kazounis E, et al A 24-week, all-oral regimen for rifampin-resistant tuberculosis. N Engl J Med 2022; 387:2331–43. 36546625 10.1056/NEJMoa2117166

[8] Guglielmetti L, Khan U, Velásquez GE, et al Bedaquiline, delamanid, linezolid, and clofazimine for rifampicin-resistant and fluoroquinolone-resistant tuberculosis (endTB-Q): an open-label, multicentre, stratified, non-inferiority, randomised, controlled, phase 3 trial. Lancet Respir Med 2025; 13:809–20. 40683298 10.1016/S2213-2600(25)00194-8 PMC13508822

[9] Guglielmetti L, Khan U, Velásquez GE, et al Oral regimens for rifampin-resistant, fluoroquinolone-susceptible tuberculosis. N Engl J Med 2025; 392:468–82. 39879593 10.1056/NEJMoa2400327 PMC7617355

[10] Mdlenyani L, Mohamed Z, Stadler JAM, et al Treatment outcomes of bedaquiline-resistant tuberculosis: a retrospective and matched cohort study. Lancet Infect Dis 2025; 25:1149–58. 40543523 10.1016/S1473-3099(25)00218-X PMC12662777

[11] Singla R, Khan S, Silsarma A, et al Bedaquiline resistance and treatment outcomes among patients with tuberculosis previously exposed to bedaquiline in India: a multicentric retrospective cohort study. Clin Infect Dis 2025; 81:846–52. 40079698 10.1093/cid/ciaf068 PMC12596394

[12] Metcalfe JZ, Weir IR, Scarsi KK, et al A 3-month clofazimine–rifapentine-containing regimen for drug-susceptible tuberculosis versus standard of care (Clo-Fast): a randomised, open-label, phase 2c clinical trial. Lancet Infect Dis 2026; 26:46–54. 40915311 10.1016/S1473-3099(25)00436-0 PMC12990188

[13] Gillespie SH, Crook AM, McHugh TD, et al Four-month moxifloxacin-based regimens for drug-sensitive tuberculosis. N Engl J Med 2014; 371:1577–87. 25196020 10.1056/NEJMoa1407426 PMC4277680

[14] Horsburgh CR Jr, Haxaire-Theeuwes M, Lienhardt C, et al Compassionate use of and expanded access to new drugs for drug-resistant tuberculosis. Int J Tuberc Lung Dis 2013; 17:146–52. 23211610 10.5588/ijtld.12.0017

[15] Perrin C, Athersuch K, Elder G, Martin M, Alsalhani A. Recently developed drugs for the treatment of drug-resistant tuberculosis: a research and development case study. BMJ Glob Health 2022; 7:e007490. 10.1136/bmjgh-2021-007490 PMC9020285 35440441

[16] Conradie F, Diacon AH, Ngubane N, et al Treatment of highly drug-resistant pulmonary tuberculosis. N Engl J Med 2020; 382:893–902. 32130813 10.1056/NEJMoa1901814 PMC6955640

[17] Cevik M, Thompson LC, Upton C, et al Bedaquiline-pretomanid-moxifloxacin-pyrazinamide for drug-sensitive and drug-resistant pulmonary tuberculosis treatment: a phase 2c, open-label, multicentre, partially randomised controlled trial. Lancet Infect Dis 2024; 24:1003–14. 38768617 10.1016/S1473-3099(24)00223-8

[18] Frick M, Dang G. The right to science: a practical tool for advancing global health equity and promoting the human rights of people with tuberculosis. In: Porsdam H, Porsdam Mann S, eds. The right to science. 1st ed. Cambridge: Cambridge University Press, 2021:246–67.

[19] Research, regulatory, and access considerations regarding pretomanid . Published online May 18, 2019. Available at: https://globaltbcab.org/statement/research-regulatory-and-access-considerations-regarding-pretomanid/. Accessed 29 July 2025.

